# Identification and functional study of a mild allele of *SlDELLA* gene conferring the potential for improved yield in tomato

**DOI:** 10.1038/s41598-018-30502-w

**Published:** 2018-08-13

**Authors:** Yoshihito Shinozaki, Kentaro Ezura, Jianhong Hu, Yoshihiro Okabe, Camille Bénard, Duyen Prodhomme, Yves Gibon, Tai-ping Sun, Hiroshi Ezura, Tohru Ariizumi

**Affiliations:** 1 Faculty of Life and Environmental Sciences, University of Tsukuba, Tsukuba, Ibaraki, 305-8572 Japan; 2 Research Fellow of Japan Society for Promotion of Science (JSPS), Kojimachi, Tokyo, 102-0083 Japan; 3 Department of Biology, Duke University, Durham, North Carolina 27708 USA; 4 UMR 1332 Biologie du Fruit et Pathologie, INRA, Univ, Bordeaux, Villenave d’Ornon F-33883 France

## Abstract

Parthenocarpy, or pollination-independent fruit set, is an attractive trait for fruit production and can be induced by increased responses to the phytohormone gibberellin (GA), which regulates diverse aspects of plant development. GA signaling in plants is negatively regulated by DELLA proteins. A loss-of-function mutant of tomato *DELLA* (*SlDELLA*), *procera* (*pro*) thus exhibits enhanced GA-response phenotypes including parthenocarpy, although the *pro* mutation also confers some disadvantages for practical breeding. This study identified a new milder hypomorphic allele of *SlDELLA*, *procera-2* (*pro-2*), which showed weaker GA-response phenotypes than *pro*. The *pro-2* mutant contains a single nucleotide substitution, corresponding to a single amino acid substitution in the SAW subdomain of the SlDELLA. Accumulation of the mutated *SlDELLA* transcripts in wild-type (WT) resulted in parthenocarpy, while introduction of intact *SlDELLA* into *pro-2* rescued mutant phenotypes. Yeast two-hybrid assays revealed that SlDELLA interacted with three tomato homologues of GID1 GA receptors with increasing affinity upon GA treatment, while their interactions were reduced by the *pro* and *pro-2* mutations. Both *pro* and *pro-2* mutants produced higher fruit yields under high temperature conditions, which were resulted from higher fruit set efficiency, demonstrating the potential for genetic parthenocarpy to improve yield under adverse environmental conditions.

## Introduction

Fruit set, the developmental transition of the ovary into fruit, is critical for determining yield in fruit-bearing crops. However, the efficiency of fruit set is often inhibited by a failure of pollination and/or fertilization due to unfavorable environmental conditions. For instance, low or high temperature stress during flower development inhibits pollen production and fertilization, leading to a critical reduction of the yield performance in tomato (*Solanum lycopersicum*)^1–3^. Parthenocarpy, the production of seedless fruit or pollination-independent fruit set, can potentially increase the efficiency of fruit set even under unfavorable conditions during reproductive phase; it is thus recognized as an attractive trait for many fruit crops to stabilize or even enhance yield performance. Despite its potential value, the adoption of parthenocarpy has nevertheless been limited to only some fruit crops including tomato, as it is usually accompanied with unfavorable traits, such as reduced fruit quality^4–6^. Therefore, the acquisition of novel genetic parthenocarpic resources without such unfavorable traits as well as understanding the molecular mechanism underlying parthenocarpy would broaden the possibility to breed novel parthenocarpic varieties.

There are a number of unambiguous pieces of evidence pointing to plant hormones such as auxin, gibberellin (GA) and cytokinin as positive regulators of fruit set initiation^7–9^. Indeed, the ovaries of naturally parthenocarpic tomato mutants including *pat, pat-2* and *pat-3/pat-4* contain high amounts of GAs^10–12^. GA is a tetracyclic diterpenoid that regulates diverse aspects of plant development including stem elongation, seed germination, the transition to flowering and fruit set; and a large body of evidence has demonstrated that the degree of GA responses results from the coordination between GA metabolism and its signaling pathway^13^. In higher plants, the GA signaling pathway is activated by the degradation of a negative regulator of GA signaling, known as DELLA, through the ubiquitin 26S proteasome pathway, thus triggering GA responses^14^. The DELLA protein consists of an N-terminal DELLA regulatory domain that is important for binding to the GIBBERELLIN INSENSITIVE DWARF 1 (GID1) GA receptors; and a C-terminal GRAS domain that is suggested to function in the repression of GA responses by interacting with downstream components^14,15^. The GRAS domain further consists of several conserved subdomains including LHR1, VHIID, LHR2, PFYRE and SAW; mutations in the GRAS domain often result in loss-of-function of DELLA that causes enhanced GA phenotypes. One exceptional gain-of-function mutation in the rice *DELLA* (*slr1-d4*) diminishes the stability of the GID1-GA-DELLA complex but retains the suppressor activity^16^.

In tomato, *PROCERA*/*SlDELLA* is believed to be the solo *DELLA* gene, and the loss-of-function *procera* (*pro*) mutation corresponding to a single nonsynonymous substitution in the GRAS domain of the SlDELLA displays enhanced GA phenotypes including parthenocarpy^17,18^. Therefore, this mutation is the subject of much attention in studies seeking to explore the mechanism of fruit set, as well as for breeding purposes. However, the *pro* mutant also shows some disadvantages for such a breeding program, such as elongated stem, reduction of flower number and an increase in malformed fruit production. Recently, other loss-of-function mutants of *SlDELLA* produced through targeted mutagenesis or transposon-based mutagenized populations were reported; those mutants showed even more severe phenotypes than *pro*^19,20^. In this study, we identified a new, milder hypomorphic allele of tomato *DELLA*, designated *pro-2*, whose mutation is located in the SAW subdomain of the GRAS domain. The *pro-2* plants showed a potential for high yield performance in optimal (climate-controlled) and unfavorable reproductive conditions with less qualitative fruiting drawbacks compared to *pro*. Both *pro* and *pro-2* mutations led to reduced interaction with homologues of the GID1 GA receptors, while the impact varied depending on the counterpart GID1 family members. These results indicate that the newly identified *pro-2* mutant can be a candidate breeding resource for parthenocarpic varieties as well as a useful tool to analyze functional associations of tomato DELLA and specific GID1 family members in the GA response in developmental processes including fruit set.

## Results

### Identification of a new recessive mutant allelic to *procera*

**Figure 1:**
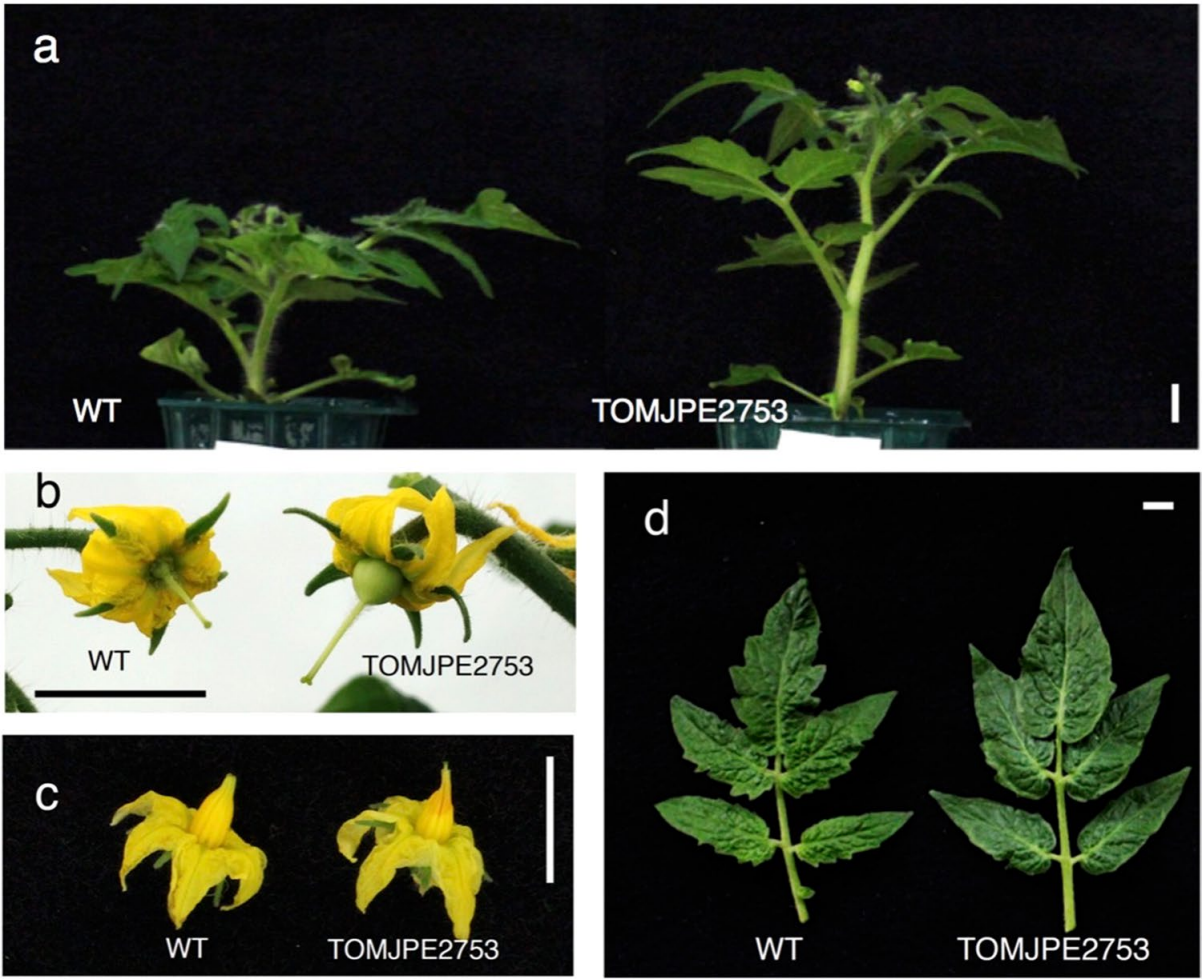
Characteristic mutant phenotypes in the TOMJPE2753 line. (**a**) Six-week-old plants. (**b**) Parthenocarpic fruit initiation from emasculated flower in TOMJPE2753 at 4 days after anthesis. (**c**) Flowers with stamen and style. (**d**) Leaflets of the 6th node. Bars = 1 cm.

**Figure 2:**
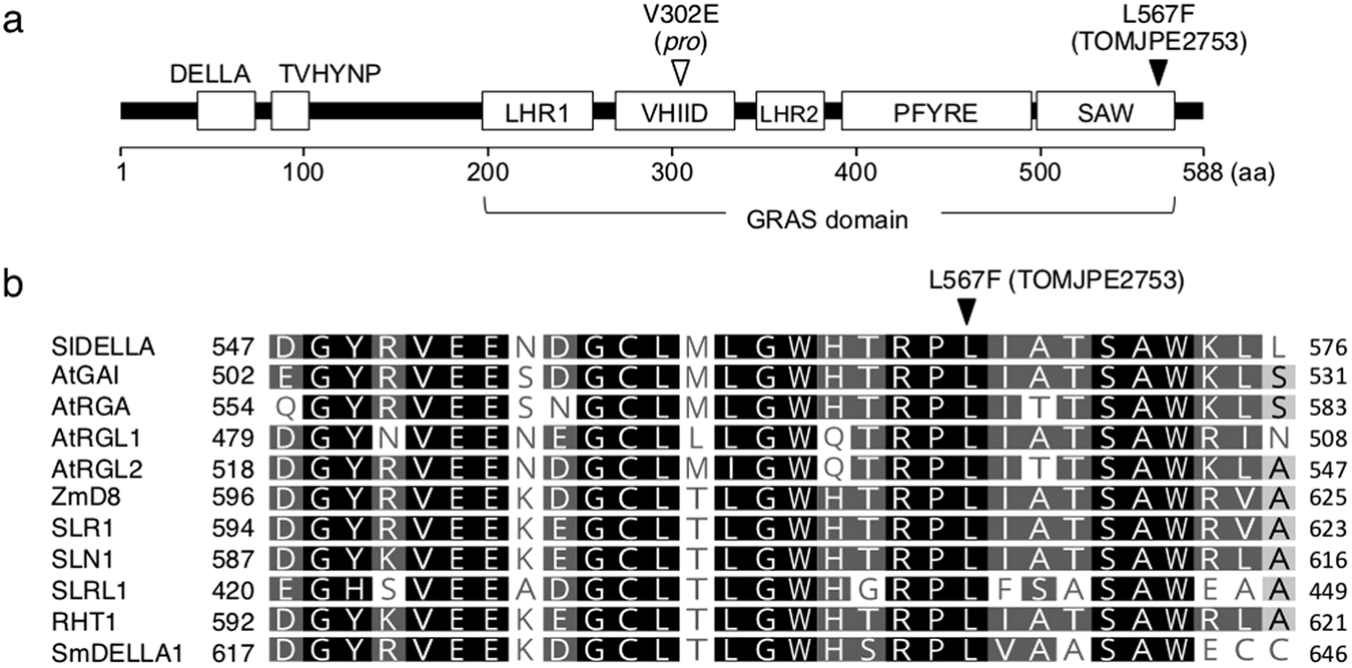
A mutation in the SlDELLA SAW subdomain of the TOMJPE2753. (**a**) Schematic structure of SlDELLA. Each subdomain is indicated by white box. An L567F substitution in the TOMJPE2753 is indicated by black arrow head. The mutation site of a classic *Sldella* mutant, *pro*, in the VHIID subdomain is indicated by white arrow head. (**b**) Sequence comparison of the C terminus region of DELLA proteins from various plant species including *Arabidopsis thaliana* (AtGAI, AtRGA, At RGL1 and AtRGL2), *Zea mays* (ZmD8), *Oryza sativa* (SLR1, SLN1 and SLRL1), *Triticum aestivum* (RHT1) and *Selaginella moellendorffii* (SmDELLA1).

We have previously produced a large-scale collection of Micro-Tom mutagenized populations^21,22^. From the ethyl methanesulfonate (EMS)-treated mutagenized M_2_ populations grown in the greenhouse, a visual screening found a mutant (TOMJPE2753) exhibiting greater plant height than wild-type (WT) and parthenocarpy (Fig. 1a,b). This mutant produced flowers that contained an elongated style protruding from the anther (Fig. 1c), and leaflets with smoother margins (Fig. 1d) compared to the WT. These vegetative and reproductive phenotypes resembled those of a constitutive GA response mutant, *procera* (*pro*), conferred by a loss-of-function of *SlDELLA*^17,18^. We thus directly sequenced the *SlDELLA* gene in the TOMJPE2753 mutant and found a C to T single nucleotide transition, known as a major nucleotide substitution induced by EMS^23^, at the 1699th nucleotide (c1699t) from the translational start site. This transition results in a nonsynonymous amino acid mutation from Leu (L) to Phe (F) at the 567th position (L567F) in the SAW subdomain of the GRAS domain (Fig. 2a). The L567 in the SlDELLA corresponds to a highly conserved amino acid residue among homologous DELLA proteins of various species (Fig. 2b).

To examine whether the identified TOMJPE2753 mutant is allelic to the known *pro* mutant, allelism tests were conducted for the reproductive phenotypes in the greenhouse. Both TOMJPE2753 and *pro* in the Micro-Tom background produced flowers with elongated and protruding styles and parthenocarpic fruit (Supplementary Fig. S1). When the TOMJPE2753 was crossed with the *pro* mutant, the F_1_ plants also showed these phenotypes. The F_1_ progenies between WT and TOMJPE2753 or *pro* produced normal flowers (Supplementary Fig. S1a). The TOMJPE2753 and *pro* both showed high parthenocarpic rates of 75% and 81%, respectively (Supplementary Fig. S1b). The F_1_ plants between TOMJPE2753 and *pro* (TOMJPE2753 × *pro*) showed a high rate of parthenocarpic fruit formation (90%). Although WT and F_1_ plants between WT and TOMJPE2753 (TOMJPE2753 × WT) or *pro* (*pro* × WT) showed certain proportions of parthenocarpy (5, 8 and 20%, respectively) under our greenhouse condition, those values were clearly lower than the TOMJPE2753, *pro* or their F_1_ progeny (TOMJPE2753 × *pro*). These results revealed that the TOMJPE2753 and *pro* mutants are allelic, and that the mutation in TOMJPE2753 is recessive. Analysis of additional phenotypes of the TOMJPE2753 × WT F_1_ plants, including plant height and leaf shape (Supplementary Fig. S2), further supports that TOMJPE2753 is a recessive mutant.

The *pro* has been identified as a monogenic recessive mutant^17^. To further understand the genetic mode of inheritance of the allelic TOMJPE2753 mutant, qualitative and quantitative mutant phenotypes and their linkage with the c1699t mutation were investigated under greenhouse conditions. In the F_2_ progeny of TOMJPE2753 backcrossed with the WT, the segregation ratio of normal and mutant phenotype for the flower morphology was 31:13, which corresponded to the expected 3:1 for a single recessive gene at the 5% level by the chi-square test (χ^2^ = 0.485). A derived cleaved amplified polymorphic sequence (dCAPS) marker was designed to genotype the WT and c1699t mutation allele in the *SlDELLA*. The normal and mutant flower phenotypes were co-segregated in the F_2_ population with the homozygous WT or heterozygous alleles and the homozygous mutant allele of *SlDELLA*, respectively (data not shown). The averaged values of the quantitative plant height and parthenocarpic rate traits were compared among different genotypes. In the F_2_ progeny, the heterozygous plants exhibited a comparable height (Supplementary Table S1) and parthenocarpic rate (Supplementary Table S2) to plants with homozygous WT allele or the original Micro-Tom WT plants, while homozygous mutant plants showed significantly greater plant height and parthenocarpic rate. These results indicate that the monogenic recessive mutation in *SlDELLA* gene was tightly linked with vegetative and reproductive phenotypic alteration.

### Transgenic complementation and induction of mutant phenotypes observed in TOMJPE2753

**Figure 3:**
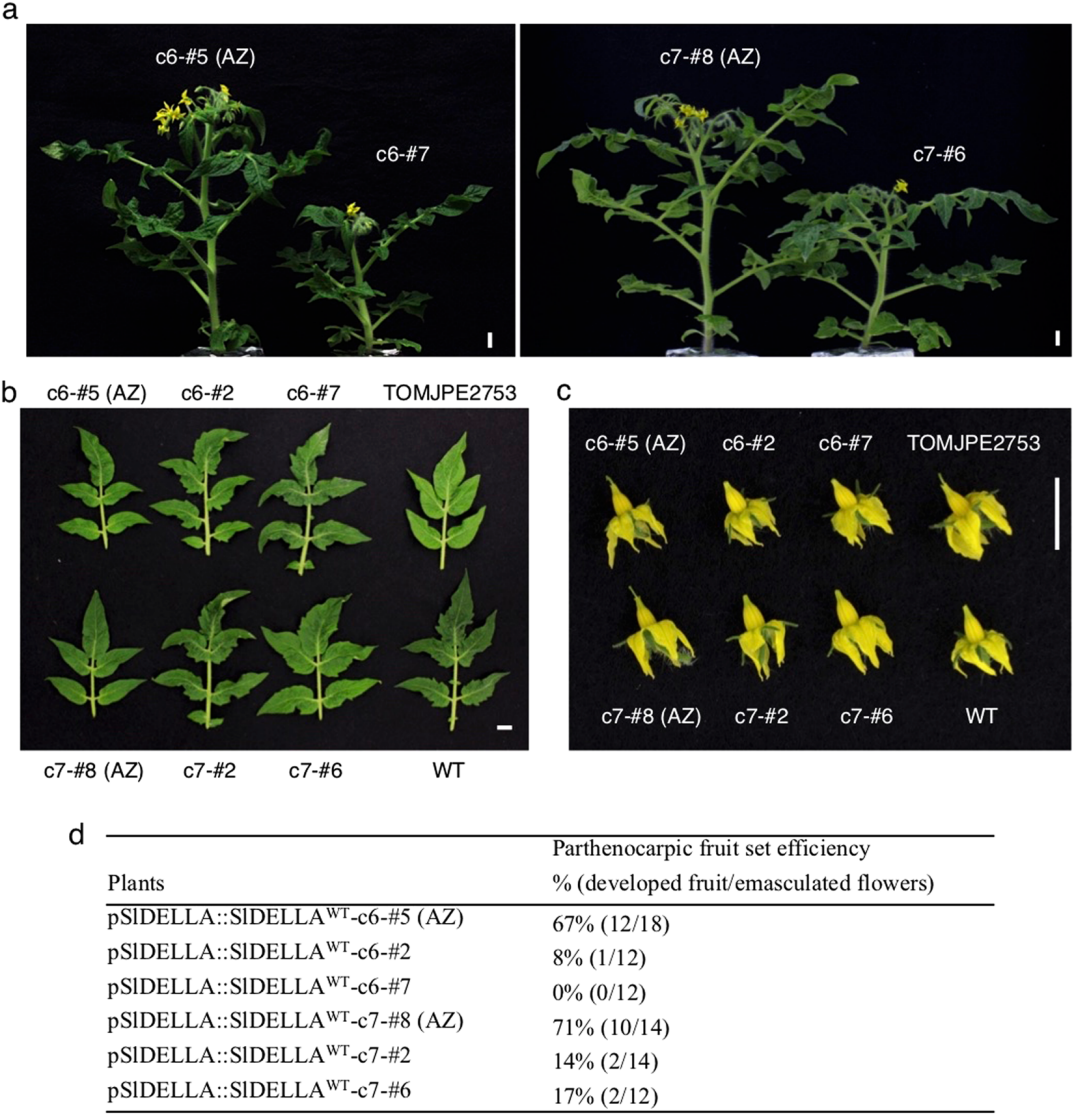
Effect of *SlDELLA* expression on vegetative and reproductive development in TOMJPE2753 background. Representative pictures of (**a**) eight-week-old plants, (**b**) leaflets of the 6th node and (**c**) flowers of transgenic and nontransgenic azygous plants. (**d**) Parthenocarpic rate in transgenic lines. Two transgenic plants (c6-#2 and c6-#7) from line c6 and two transgenic plants (c7-#2 and c7-#6) from line c7 were used along with their corresponding azygous siblings c6-#5 and c7-#8, respectively. AZ, azygous. Bars = 1 cm.

To confirm that the TOMJPE2753 is a new *Sldella* mutant, complementation rescue of the mutant phenotypes was conducted by introducing the WT *SlDELLA* under the control of its native promoter (pSlDELLA::SlDELLA^WT^) into TOMJPE2753 mutant plants. Initial screening at the T_0_ generation found that the plant heights of nine out of ten independent transgenic lines were normal, and three of them barely produced parthenocarpic fruit. Two screened independent lines, c6 and c7, with normal height and fruit production were selected for further analysis at the T_2_ generation. Compared to the azygous (AZ) sibling plants (c6-#5 and c7-#8) that do not carry transgene, morphological phenotypes, including plant height (Fig. 3a), leaf shape (Fig. 3b) and stylar length (Fig. 3c), in transgenic plants of both lines were restored and comparable with those of WT. The transgenic plants showed largely reduced parthenocarpic ability compared to the corresponding nontransgenic AZ plants in the TOMJPE2753 mutant background (Fig. 3d). These results demonstrate that SlDELLA is responsible for the mutant phenotypes in TOMJPE2753.

In rice, the DELLA protein forms a homodimer and the overexpression of a loss-of-function DELLA with a domain required for dimerization but lacking the GRAS domain exerts a dominant-negative effect and makes rice plants slender due to increased GA sensitivity^24^. Since the TOMJPE2753 mutation locates within the GRAS domain, the mutant form (*SlDELLA*^*L567F*^) under the control of the 35S promoter (p35S::SlDELLA^L567F^) was introduced into the WT background plants to assess its dominant-negative effect. We generated three independent transgenic lines that showed parthenocarpy and two representative transgenic lines, m2 and m4, both of which were confirmed to express the mutated *SlDELLA*^*L567F*^ transcripts and were thus selected for further analysis (Supplementary Fig. S3a). The transgenic plants carrying the transformation construct displayed mutant phenotypes observed in TOMJPE2753, including elongated plant stature (Supplementary Fig. S3b), attenuation of the serrated edge of the leaf (Supplementary Fig. S3c), longer style (Supplementary Fig. S3d) and parthenocarpic fruit formation (Supplementary Fig. S3e and f). These results indicate that ectopically expressed *SlDELLA*^*L567F*^ can confer constitutive GA response phenotypes including parthenocarpy. Most likely, this is via an underlying dominant-negative mechanism that may mask normal function of endogenous SlDELLA.

### Phenotypic comparison of the identified *pro-2* with WT and *pro* mutant

**Figure 4:**
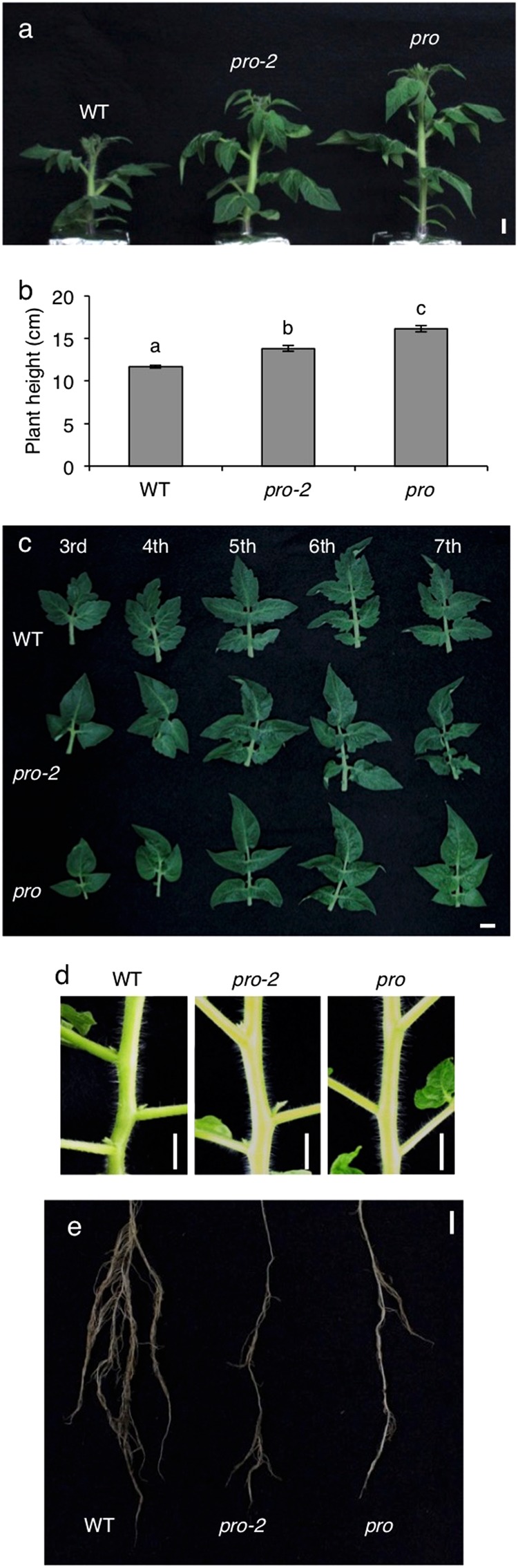
Vegetative and root phenotypes of *pro-2* compared with WT and *pro*. (**a**) Representative 6-week-old plants. (**b**) Plant height at 6 weeks old. Values are mean ± SE (n = 5). Different letters indicate significant differences (*P* < 0.05, Tukey–Kramer test). (**c**) Representative leaves of 3rd to 7th node from cotyledons at 8 weeks old. (**d**) Representative axillary buds of 4th to 6th node at 9 weeks old. (**e**) Representative root growth at 6 weeks old. Scale bars = 1 cm.

The M_3_ population of the TOMJPE2753 mutant was backcrossed to WT Micro-Tom, followed by two self-pollinations, to eliminate the mutagen-induced background mutations and obtain a BC_1_S_2_ population harboring homozygous mutated *SlDELLA*, renamed here as *procera-2* (*pro-2*), which was used for a further phenotypic comparison with WT and the *pro* mutant. The comparison of the vegetative development revealed that the *pro-2* mutant was intermediate in height between the WT and *pro* mutant (Fig. 4a and b). The leaf margin of *pro-2* was smoother compared to WT, but the phenotypic change was less severe than *pro* that completely lacked the serrated edge (Fig. 4c). It has been shown that the branching architecture is altered in *pro*, in which growth of axillary buds is highly repressed^17,25^. We found that the *pro-2* produced axillary buds that grow to normal branches, which was barely found in *pro*, particularly at far nodes from apex (Fig. 4d). It has been shown that GA overproduction or increased GA signaling suppresses root growth, particularly the lateral roots, through interactions with auxin and other hormones in *Populus*^26^. Such a side effect was clearly observed in both *pro* and *pro-2* (Fig. 4e).

**Figure 5:**
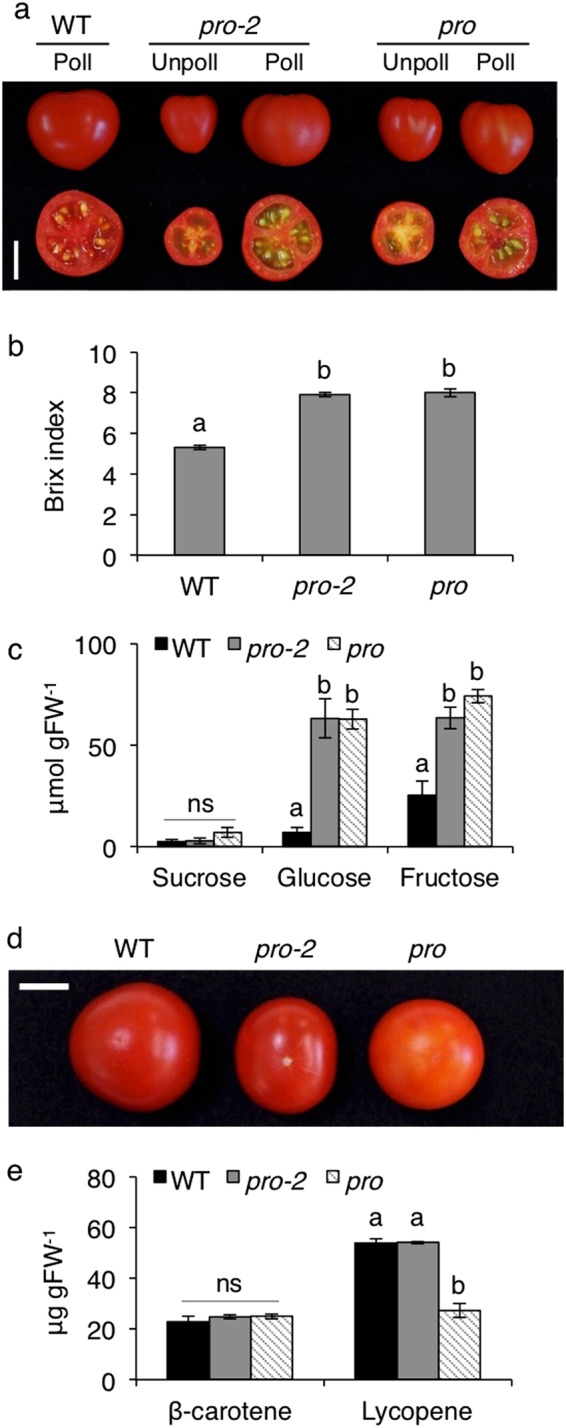
Fruit phenotypes of WT, *pro-2* and *pro*. (**a**) Representative unpollinated and manually pollinated fruit. Comparison of (**b**) Brix value and (**c**) sugar contents. (**d**) Representative orange patched *pro* fruit. (**e**) Comparison of carotenoid contents. Values are mean ± SE of (b) 20, (**c**) 3 and (**e**) 5 fruit. Different letters indicate significant differences (*P* < 0.05; Tukey–Kramer test). Poll, pollinated; Unpoll, unpollinated; ns, not significant. Scale bars = 1 cm.

Comparison of reproductive phenotypes in the optimal growth condition (climate controlled condition) showed that both *Sldella* mutants produced more leaves before the first inflorescence than WT (Table 1). On the other hand, the *pro* mutant produces a decreased number of flowers in a truss^18^, which was confirmed in our study (Table 1). The *pro-2* also produced fewer flowers per truss than WT, but more than that in *pro*. Under spontaneous self-pollination condition, both *pro and pro-2* produced more fruit than WT, which most likely resulted from higher fruit set rate, while most of the fruits were smaller and seedless, i.e. parthenocarpic (Table 1 and Fig. 5a). It is interesting, however, that *pro-2* exhibited significantly higher yield than *pro*, resulting from higher number of fruits. Although *pro* and *pro-2* displayed efficient parthenocarpy, these mutants also produced normal seeded fruit when they were manually pollinated (Fig. 5a), although the number of seeds were reduced to approximately half of WT (Table 1). Almost all fruits spontaneously produced by *pro* and *pro-2* were seedless, most likely due to a failure in self-pollination due to the protruded stigma from the anther.

**Table 1:** Comparison of reproductive phenotypes among WT, *pro-2* and *pro* in the optimal condition.

	WT	*pro-2*	*pro*
Leaves to first inflorescence (n)^z^	8.4 ± 0.3a	10.2 ± 0.2b	9.8 ± 0.2b
Flowers in the first two inflorescences (n)^z^	14.4 ± 1.0a	12.0 ± 0.4b	9.6 ± 0.5c
Fruit per plant (n)^z^	10.0 ± 0.3a	32.6 ± 0.7c	23.8 ± 1.8b
Fruit weight (g)^y^	4.8 ± 1.5a	1.9 ± 0.2b	2.1 ± 0.2b
Fruit production (g per plant)^z^	44.6 ± 2.7a	61.2 ± 3.1b	38.7 ± 3.2a
Seedless fruit (%)^y^	0	89.1	100
Seeds per manually pollinated fruit (n)^x^	43.1 ± 5.6a	19.7 ± 5.1b	22.2 ± 4.6b

^z^Ten plants. ^y^Thirty to fifty fruit. ^x^Ten fruit. Values are mean ± SE. Different letters indicate significant differences (*P* < 0.05; Tukey–Kramer test). Pollination occurred through spontaneous self-pollination.

A previous study showed that the Brix value, the indicative of levels of soluble solids content, of *pro* mature fruit is higher than that in WT^18^. We found that mature parthenocarpic fruit produced by both *pro-2* and *pro* mutants had higher Brix values than seeded WT fruit (Fig. 5a,b). The quantitative analysis of sucrose, fructose and glucose in mature red fruit revealed that contents of glucose and fructose in these mutants were nine times and two to three times higher, respectively, than in WT (Fig. 5c). However, the sucrose content was comparable among the three genotypes, suggesting that the high Brix value was derived from high hexose content. In addition, we found that *pro* often produced mature fruit with an orange patch, which was barely observed in WT and *pro-2* (Fig. 5d). This partially orange-coloration of fruits did not appear to be associated with β-carotene levels but rather with reduced levels of lycopene compared to WT pollinated and *pro-2* parthenocarpic mature fruits that showed equivalent carotenoid contents (Fig. 5e).

### High yield potential of *Sldella* mutants during a high temperature growth condition

**Figure 6:**
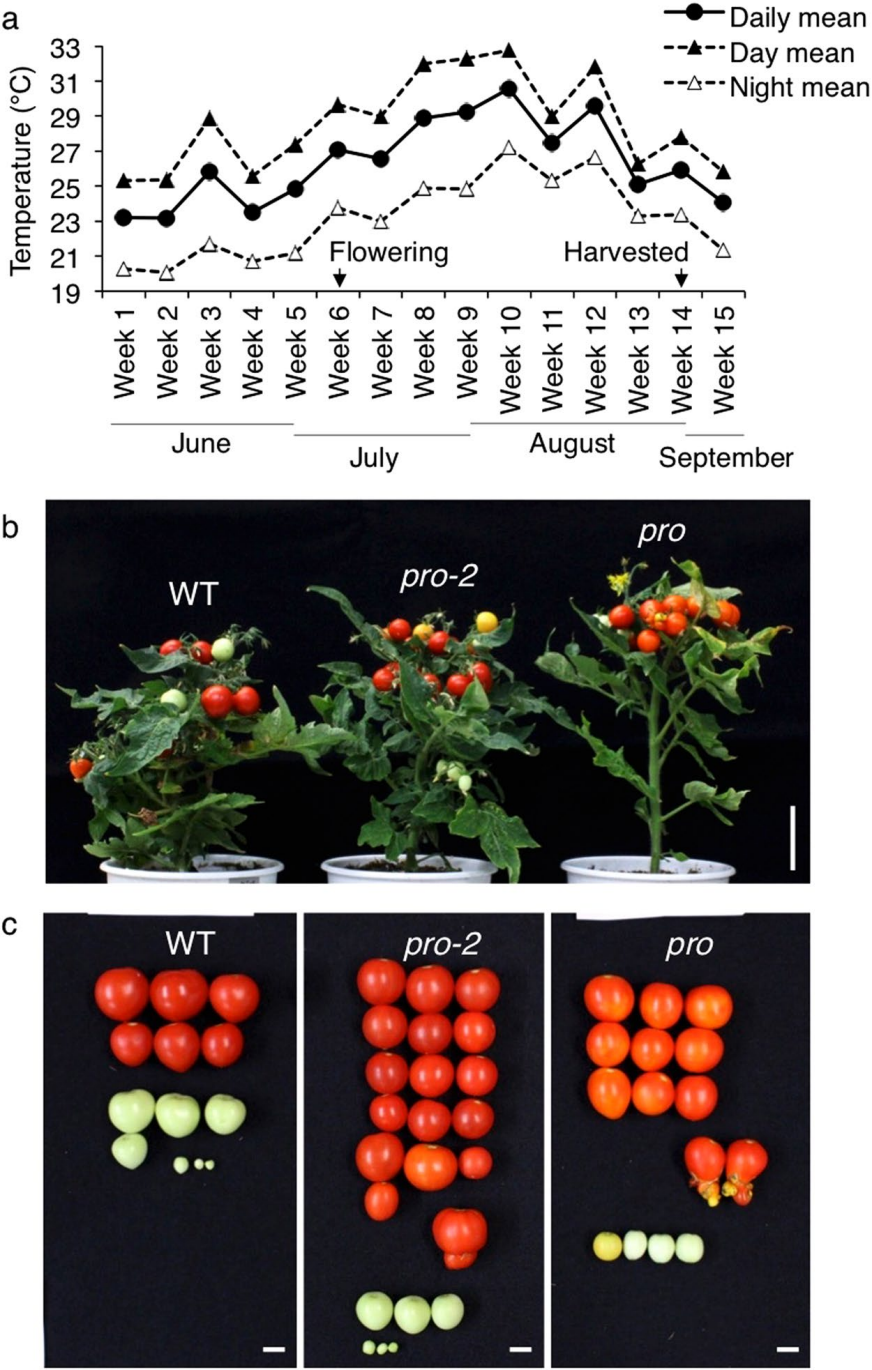
Plants and fruit of WT, *pro-2* and *pro* produced in greenhouse at high temperature. (**a**) Averaged daily, day (5 am to 7 pm) and night (7 pm to 5 am) mean temperature by each week. All plants opened the first flower during week 6 and vegetative and reproductive traits were quantified during week 14, which corresponds to during 6- and 14-week-old plant age, respectively. (**b**) Representative aerial plants at week 14. (**c**) Representative harvested fruit. Scale bars = 5 cm in panel b and 1 cm in panels c.

Since parthenocarpy is considered to be a beneficial tool for improving the yield of fruit crops under severe environmental conditions^9^, WT and two *Sldella* mutants were used to investigate productivity in the greenhouse during four months of the summer period in year 2014. One-week-old seedlings of all genotypes were planted on June 1st and then mature ripe and immature fruits were harvested during the 1st week of September. The day and night temperature variation throughout the experiment is shown in Fig. 6a. After week 6 when all plants commenced flowering, the averaged daily mean temperature remained above 27 °C for each week with the exception of week 7 and two weeks before harvesting; and remained above 29 °C at weeks 8 through 10. The averaged day and night temperature for each week ranged from 26 to 33 °C and 23 to 27 °C, respectively. The vegetative growth phenotypes observed in the optimal (climate-controlled) conditions were all reproduced in this greenhouse condition, including the attenuated axillary branching in *pro*, which led to a decrease in the fresh weight of the aerial part of the plant, and the suppression of root growth (dry weight of root) in both *Sldella* mutants (Table 2 and Fig. 6b). The total number of flowers largely varied between WT and *pro*, while that of *pro-2* was intermediate between WT and *pro*. The fruit set rate was calculated by the ratio of the number of fruits to total flowers per plant under spontaneous self-pollination condition and this was the highest in *pro* (87.9%) followed by *pro-2* (51.8%) and was much smaller in WT (10.9%). The yield of each genotype was compared by the averaged fruit production per plant (Table 2). At harvesting, the immature fruit yield was similar among WT and *Sldella* mutants. Clearly, fruit formation in WT was severely suppressed and the average yield of the mature fruit was limited to 24.3 ± 2.9 g per plant, due to a failure in fruit set under the high temperature stress, although cultivation of WT under optimal conditions produced 44.6 ± 2.7 g per plant. The average yield in both *Sldella* mutants was 1.9 (*pro*) to 2.5 times (*pro-2*) higher than in WT; it was not associated with fruit weight but with increased number of fruit per plant. The *pro-2* produced more fruit than *pro*, which was again most likely due to the less severe attenuation of axillary branching and flower production. The percentages of seedless fruit production by *pro* and *pro-2* were 100% and 99.1%, respectively (Table 2). Unexpectedly, WT also produced seedless fruit under heat stress, but the ratio was much lower than *Sldella* mutants.

**Table 2:** Vegetative and reproductive production of WT, *pro-2* and *pro* in the greenhouse during summer.

	WT	*pro-2*	*pro*
Plant height (cm)	11.8 ± 0.3a	15.2 ± 0.5b	17.9 ± 0.8c
Leaves to first inflorescence (n)	8.0 ± 0.0a	9.8 ± 0.4b	9.3 ± 0.3b
Fresh weight of aerial part (g)	46.1 ± 3.9a	38.3 ± 3.2a	27.7 ± 1.7b
Dry weight of root (g)	0.39 ± 0.06a	0.27 ± 0.03b	0.23 ± 0.02b
Flowers per plant (n)	95.0 ± 8.5a	52.2 ± 5.5b	23.5 ± 2.2c
Fruit set rate (%)	10.9 ± 1.8a	51.8 ± 1.9b	87.9 ± 4.7c
Mature fruit^z^			
Fruits per plant (n)	4.4 ± 0.6a	18.6 ± 1.2c	11.8 ± 2.0b
Fruit weight (g)	5.6 ± 0.3a	3.3 ± 0.3b	3.9 ± 0.2b
Fruit production (g per plant)	24.3 ± 2.9a	61.1 ± 2.8c	45.5 ± 5.2b
Seedless fruit (%)	20.0	99.1	100.0
Immature fruit			
Fruits per plant (n)	5.6 ± 0.7ns	7.4 ± 2.6ns	6.4 ± 2.3ns
Fruit production (g per plant)	10.9 ± 3.0ns	8.8 ± 2.9ns	10.4 ± 4.2ns
Malformed fruit			
Fruits per plant (n)	0.0 ± 0.0a	1.0 ± 0.3b	3.2 ± 0.6c
Malformed fruit per mature fruit (%)	–	5.1^y^	16.9^x^

^z^Normal shape fruit fully or partially turned to red. ^y^Five out of ninety-eight fruit.

^x^Twelve out of seventy-one fruit. All data were obtained from five plants of each genotype. Values are mean with or without ±SE. Different letters indicate significant differences (*P* < 0.05; Tukey–Kramer test). ns, not significant. Pollination occurred through spontaneous self-pollination.

One of the drawbacks of the use of *pro* for practical breeding is the production of malformed fruit with a bubbling structure at the tip of the fruit (Fig. 6c). Although both *Sldella* mutants produced such malformed fruit, its production rate was attenuated in the *pro-2* mutant (5.1%) compared to the *pro* mutant (16.9%) (Table 2). In addition, most of the *pro* mature fruits have faded orange color (Fig. 6c). Such fruit was also found in *pro-2* but with much less frequency, and barely found in WT (Fig. 6c).

### Seedling growth of *pro-2* showed moderate resistance to paclobutrazol

The loss-of-function of *DELLA* results in enhanced responses to endogenous GA and a decreasing sensitivity to the GA biosynthesis inhibitor paclobutrazol (PAC)^18,20^. To compare sensitivities to PAC, 3-day-old germinated seedlings of WT, *pro-2* and *pro* were grown in MS medium with different concentrations of PAC (0, 1 and 10 µM) for 10 days, and then the lengths of the root and shoot were measured (Supplementary Fig. S4). Although the 1 µM PAC significantly inhibited the root growth in WT, its inhibitory effect was not observed in *pro-2* and *pro* mutants. The 10 µM PAC treatment decreased the root length to a similar extent in all genotypes (Supplementary Fig. S4a). In WT, the shoot length of seedlings was also significantly decreased by 42% at the 1 µM PAC treatment compared to the control 0 µM PAC condition and no further reduction was found with 10 µM PAC. In contrast, the 1 µM PAC treatment decreased the shoot length of *pro-2* and *pro* by 22% and 10%, respectively, compared to the control condition. The 10 µM PAC treatment further inhibited their shoot growth, while the shoot length in *pro* was taller than *pro-2* seedlings grown with 1 or 10 µM PAC (Supplementary Fig. S4b). These results indicate that the seedling growth rates of these *Sldella* mutants were more resistant to PAC than WT, and that the *pro-2* mutant showed intermediate sensitivity to the PAC between the WT and *pro*.

### *pro-2* and *pro* mutant proteins impact their interactions with SlGID1 family members

Previous studies showed that GA-dependent protein interaction between the DELLA domain and the GA receptor GID1 is further stabilized by the GRAS domain of DELLA^16,27^. We used yeast two-hybrid (Y2H) assays to investigate the effect of the SlDELLA mutations of *pro-2* and *pro* on the basis of the interactions between SlDELLA and GID1 proteins. In the tomato genome, three GID1 family genes, consisting of two GID1b group members (*SlGID1b-1* [Solyc09g074270] and *SlGID1b-2* [Solyc06g008870]) and one GID1ac group member (*SlGID1ac* [Solyc01g098390]) have been identified^28^ (Supplementary Fig. S5a). We confirmed that these SlGID1s possess most of conserved amino acids essential for binding to GA or DELLA proteins^29,30^, suggesting that they are functional GA receptors (Supplementary Fig. S5b). Expression data based on transcriptome profiling of samples from various tissues^31–33^ showed that *SlGID1b-1* and *SlGID1b-2* exhibit higher expression levels than *SlGID1ac* across most of the tissues, including pistils/ovaries and young fruit (Supplementary Fig. S6).

**Figure 7:**
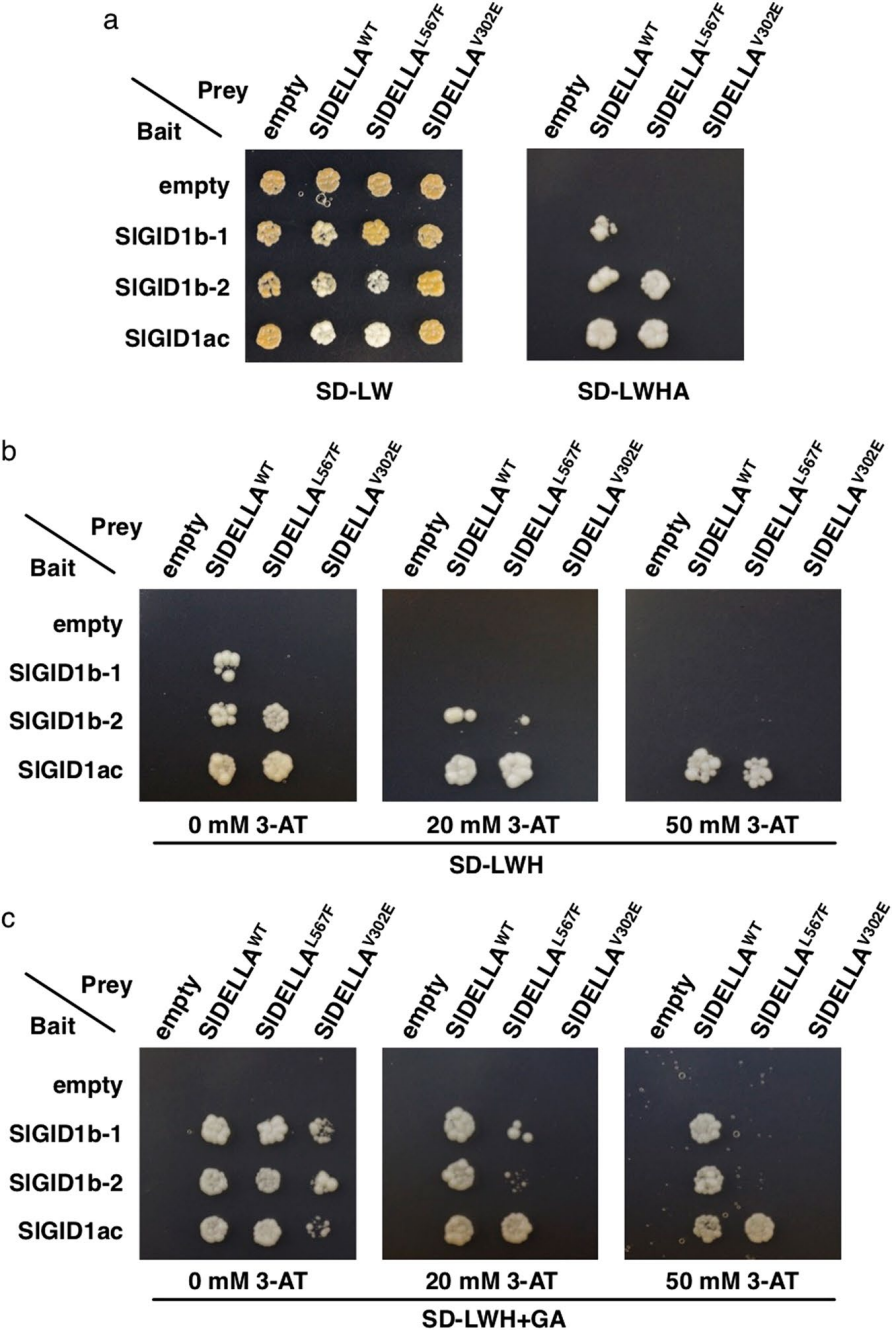
Effect of *pro-2* and *pro* mutations in SlDELLA on interactions between SlDELLA and SlGID1 family members in yeast two-hybrid assays. (**a**) Growth of yeast strain PJ69-4A transformants on SD-LW (control) and SD-LWHA plates. The growth status of PJ69-4A transformants was also observed on SD-LWH plates containing different concentrations of 3-amino-1,2,4-triazole (3-AT) without (**b**) or with (**c**) 100 μM GA_3_. SlGID1 family members were used as bait, and SlDELLA and its mutants were used as prey. Pictures of plates incubated for 5 days at 30 °C are shown.

The full-length SlDELLA protein was strongly auto-activated when it was fused with the GAL4 DNA-binding domain as a bait, which is consistent with previous reports in other plant species^34^ (Supplementary Fig. S7). SlDELLA and its mutant forms were thus fused with the GAL4 activating domain and used as a prey, and the three full-length SlGID1 proteins, SlGID1b-1, SlGID1b-2 and SlGID1ac, were used as bait in this study. The Y2H assay indicated that the normal SlDELLA (SlDELLA^WT^) could interact with all of these GID1 proteins, while the affinity varied depending on the combinations (Fig. 7). In the absence of GA, the yeast transformed with SlDELLA^WT^ and each of the SlGID1s could grow in moderate selection medium without 3-AT application (Fig. 7a and b). SlGID1ac appeared to show the strongest interaction with the SlDELLA^WT^ since the SlGID1ac-transformed yeast grew even under severely selective conditions (50 mM 3-AT), while the growth of yeast transformed with SlGID1b-1 or SlGID1b-2 was inhibited by the application of 3-AT (Fig. 7b). SlDELLA^L567F^ corresponding to the *pro-2* mutation could interact with SlGID1b-2 or SlGIDac, while SlDELLA^V302E^ corresponding to the *pro* mutation did not show any interaction with SlGID1s.

In the presence of GA, the interaction of SlGID1b-1 or SlGID1b-2 with SlDELLA^WT^ was strengthened, as indicated by strong yeast growth even with 20 and 50 mM 3-AT application, while such a positive effect was not clearly evident for SlGID1ac compared with SlGID1b-1 or SlGID1b-2 (Fig. 7c). GA application enabled the SlDELLA^L567F^ to interact with SlGID1b-1 in the absence of 3-AT, although their interaction was weaker than the SlDELLA^WT^-SlGID1b-1 interaction since the growth of yeast transformed with SlDELLA^L567F^ and SlGID1b-1 was inhibited in the presence of 3-AT, unlike SlDELLA^WT^. Furthermore, the positive effect of GA application was not observed for the interaction between SlDELLA^L567F^ and SlGID1b-2 or SlGID1ac. GA application promoted the interactions between SlDELLA^V302E^ and the three SlGID1s but only under the condition without 3-AT application (Fig. 7c). It is of note that SlDELLA^L567F^ interacted with all SlGID1, although SlDELLA^V302E^ did not interact with any SlGID1 under the condition of 20 mM 3-AT in the presence of GA. These results suggested that both SlDELLA^L567F^ and SlDELLA^V302E^ reduced the capacity to interact with the three SlGID1s compared to SlDELLA^WT^; and SlDELLA^L567F^ attained a higher affinity with the three SlGID1 than SlDELLA^V302E^.

## Discussion

### *pro-2* is a novel and mild hypomorphic allele of *SlDELLA* gene

This study identified *pro-2*, a new mutant tomato allele of the GA signaling component, DELLA, whose mutation was localized in the SAW subdomain within the GRAS domain. Previous studies demonstrated that mutations in the SAW subdomain of rice DELLA (SLR1) lead to either reduced (*slr1–2*, *slr1–3*, *slr1–4* and *slr1–7*) or increased (*slr-d4*) repression activity depending on the site and/or type of mutation^16,35,36^. The *pro-2* mutant harbored a single amino acid substitution at the 567th Leu that is highly conserved within the DELLA proteins of plant species (Fig. 2b); and this conferred enhanced GA phenotypes similar to previously reported loss-of-function mutants of *SlDELLA* such as *pro*, *pro*^*TALEN_2*^, *pro*^*ΔGRAS*^ and *SlDELLA*-silenced plants using an antisense strategy^17,19,20,37^. This study demonstrated that the transgenic tomato plants in which the mutated *SlDELLA*^*L567F*^ transcripts were expressed showed enhanced GA phenotypes including increased stem elongation, reduced leaf serration, stigma protrusion and parthenocarpy, as observed in the *pro-2* mutant (Supplementary Fig. S3). Furthermore, introduction of the full length of the *SlDELLA* coding sequence under the control of the native promoter fully rescued the *pro-2* phenotypes (Fig. 3). Therefore, the conserved Leu plays an important role for conferring repression activity of SlDELLA and its substitution to Phe was responsible for *pro-2* mutant phenotypes.

The *pro-*2 mutant displayed milder GA-response phenotypes except for root growth, compared to the *pro* mutant (Fig. 4 and Table 1). Additionally, the *pro-2* seedling was more sensitive to a GA biosynthesis inhibitor, PAC, compared to the *pro* seedlings as indicated by the shoot growth response (Supplementary Fig. S4). It has been previously reported that *pro*^*ΔGRAS*^ or *pro*^*TALEN_2*^, which completely lack the GRAS domain, show exaggerated GA-induced phenotypes as well as nearly complete resistance to PAC^17,20^. These and our results suggest that the *pro-2* mutant is a novel weak hypomorphic *Sldella* allele.

### *pro-2* may reduce stabilization of the GA-GID1-DELLA complex

In the current model, the molecular mechanism of de-repression of the DELLA inhibitory effect is mediated through the reception of GA by GID1 followed by the formation of the GID1-GA-DELLA complex, which is subsequently recognized by the E3 ubiquitin ligase F-box protein that marks DELLA with ubiquitination and subsequent degradation, hence inducing the GA responses^14^. However, the role of the SAW subdomain in the GRAS domain’s repressor-related activity and interaction with GID1 is poorly understood in tomato.

Previously, Hirano *et al*.^16^ showed that an amino acid substitution of rice DELLA (SLR1), from Gly to Val at position 576, in the SAW subdomain reduced its interaction capacity with GID1 (OsGID1)-GA without affecting the repressor activity of SLR1, while the substitution of the Leu-Phe at positions 589–590 to Ala-Ala reduced both the affinity with GID1-GA and the repressor activity. In contrast, artificial residue substitutions by an Ala scanning experiment at another seven conserved positions in the SAW subdomain, including Arg at position 621 that corresponds to just two residues upstream from the *pro-2* mutation, did not influence the OsGID1-GA-SLR1 interaction^16^, implying that the important amino acids in the SAW subdomain that stabilize the interaction are not widely scattered, but rather specifically localized. Our study reveals that the substitution of a highly conserved Leu at position 567 (L567) in the SAW subdomain to Phe (SlDELLA^L567F^) which corresponds to the *pro-2* weak loss-of-function mutation, caused the reduction of the GA-independent and -dependent affinity of SlDELLA with two GID1 homologues, SlGID1b-1 and SlGID1b-2 (Fig. 7c). Furthermore, we found that SlDELLA^V302E^, which corresponds to the *pro* mutation located in the VHIID subdomain, also caused both the attenuation of the repressor activity and the reduction of the affinity with SlGID1 proteins. These data suggest that the highly conserved Val at the 302nd amino acid in the VHIID subdomain and the Leu at the 567th amino acid in SAW subdomain, both play important bifunctional roles in the stabilization of the DELLA-GA-GID1 complex and the repressor activity of DELLA.

This study also showed strong GA-independent interaction between WT SlDELLA and a GID1ac-group member (SlGID1ac), even under highly stringent conditions (50 mM 3-AT) (Fig. 7). Similarly, GA-independent interactions of GID1-DELLA have been reported in other species. For instance, *Arabidopsis* has two GID1ac- (AtGID1a and AtGID1c) and one GID1b-group member (AtGID1b), the latter of which can interact with DELLA proteins in the absence of GA^38^. Brassica and soybean GID1b-group members homologous to AtGID1b can interact with their own DELLA orthologues in the absence of GA, most likely through an unconserved loop region that may contribute to the stabilization of the GID1-GA interaction^39^. The present Y2H assays showed that GID1ac-group member SlGID1ac, rather than GID1b-group members, had a highly stable GA-independent interaction with SlDELLA (Fig. 7). It is possible that the strong stability of SlGID1ac-SlDELLA interaction is associated with the loop region whose sequence is well conserved between SlGID1b-1 and SlGID1b-2 but not between these and SlGID1ac (Supplementary Fig. S5b). Also, the L567F mutation did not influence the GA-independent interaction with SlGID1ac, whereas it reduced the interaction with SlGID1b-1 and SlGID1b-2 (Fig. 7). Furthermore, *SlGIDac* transcripts were much less abundant throughout tissues in tomato (Supplementary Fig. S6). These results suggested that the SlGID1b-1 and SlGID1b-2 mainly contribute to a de-repression mechanism of GA signaling.

### High yield potential of *pro-2* under heat stress condition through improved fruit set efficiency

In fruit crops, the number and weight of fruit essentially determine the yield and thus high fruit set efficiency is an important factor to achieve high yield performance. Since harsh temperature conditions often cause male sterility leading to a failure in fruit set initiation, fruit set induced by parthenocarpy could be useful to improve yield under such stressed conditions^9^. It has been reported that the daily mean temperature is particularly critical for fruit set, e.g., the setting rate and number of fruit were largely decreased from 25 °C to 27 °C and further declined at 29 °C^2^. In our greenhouse experiment, the daily mean temperature averaged by week remained above 27 °C after flowering at week 6, with the exception of week 7 and two weeks before harvesting, and above 29 °C at weeks 8 through 10. This temperature condition indeed suppressed pollination-dependent fruit production in WT, compared to the optimal condition (Tables 1 and 2). This experiment demonstrated that the two different alleles of *Sldella* mutants exhibited higher yield than WT under this natural heat stress condition (WT, 24.3 ± 2.9 g; *pro-2*, 61.1 ± 2.8 g; *pro*, 45.5 ± 5.2 g) (Table 2). Although the average fruit weight produced by *Sldella* mutants was lower than WT (WT, 5.6 ± 0.3 g; *pro-2*, 3.3 ± 0.3 g; *pro*, 3.9 ± 0.2 g), these *Sldella* mutants showed higher yield potential due to their higher fruit set rate through parthenocarpy (Table 2). This demonstrates the potential of genetic parthenocarpy under unfavorable growth conditions to enhance fruit productivity. Interestingly, although the average fruit weight produced by *pro-2* mutant was equivalent to the *pro* mutant, the normal red fruit yield of the *pro-2* mutant was higher than the *pro* mutant, which was also observed in the optimal condition (Table 1). This superior outcome of the milder *pro-2* allele might be related to its higher number of flowers per plant, which is most likely due to less severe reduction in the number of flowers per truss and/or axillary branches (Table 1 and Figs 4d and 6b), rather than the fruit set rate that was higher in *pro* than *pro-2* (Table 2), suggesting that the weaker *Sldella* allele is more suitable for a breeding program. Indeed, this study also demonstrated that *pro-2* exhibited less unfavorable fruit and vegetative characteristics than the *pro* mutant, including a decreased rate of malformed fruit formation and the attenuation of increased stem elongation (Table 2 and Fig. 4b). Additionally, *pro* occasionally produced partially orange mature fruit that is associated with less lycopene content most likely resulting from decreased sensitivity to a ripening hormone ethylene due to the antagonistic effect of GA^40^; whereas the *pro-2* mutant produced mature red fruit with the same level of lycopene accumulation as the WT (Fig. 5e). Since tomato fruit constitutes a major natural source of lycopene, considered to have a range of benefits to human health due to its strong antioxidant property^41^, the mild *pro-2* allele could be more suitable than the *pro* allelic mutant from the view point of productivity as well as quality of the fruit.

Finally, we observed that mature red fruit of both *Sldella* mutants contained higher sugar contents estimated by Brix value, most likely due to higher levels of glucose and fructose (Fig. 5b and c), which are important components for the nutritional value and for the taste of the fruit. Thus, in terms of both yield and quality (representing market value), the use of *Sldella* mutants for breeding varieties with high sugar contents is attractive.

Here we isolated a new *sldella* mutant allele *pro-2* that exhibited milder GA-induced phenotypes and a substantial potential for higher yield under heat stress condition compared to *pro*. These traits resulted from efficient parthenocarpy and most likely the attenuation of the drawbacks that accompany the severe GA responses found in *pro*. These results demonstrate the potential of the mild mutant allele for breeding of parthenocarpic tomato varieties. However, this study used cv. Micro-Tom as a genetic background which concomitantly harbors a mutation affecting brassinosteroid biosynthesis (*dwarf*) that possibly influences GA response^42^. Future study should test the effectiveness of the *pro-*2 allele in other genetic backgrounds. In addition, *pro-2* still carries undesirable traits for breeding (i.e. small size of fruit and reduced seed numbers), although a gene pyramiding strategy using QTL that increases fruit size^43^ or PAC treatment to promote seed production from parthenocarpic plants^44^ may alleviate these drawbacks.

## Methods

### Plant materials and growth conditions

This study used tomato (*Solanum lycopersicum*) cv. Micro-Tom including the WT, the *pro-2* mutant (line name TOMJPE2753) isolated from the previously developed ethyl methanesulfonate (EMS)-mutagenized populations^21,45^ and the *pro* mutant^46^. The M_3_ generation of the *pro-2* mutant line was backcrossed to Micro-Tom WT followed by two self-pollinations, resulting in BC_1_S_2_ populations. The homozygous *pro-2* allele of this population was used for phenotypic comparison experiments. Seeds were imbibed with deionized water overnight and placed on a filter paper moistened with deionized water for 4–6 d at 25 °C under 16 h light at 100 μmol m^−2^ s^−1^. For cultivation in a climate-controlled room (optimal condition), the germinated seedlings were transplanted into rockwool cubes (75 × 75 × 65 mm, Grodan Delta), except when otherwise stated, and grown with a nutrient solution with an electrical conductivity (EC) of 1.6 dS m^−1^ (Otsuka Chemical) in a photoperiod of 16 h light at 25 °C (light)/20 °C (dark) under fluorescent lights at 150–200 μmol m^−2^ s^−1^. To observe the roots grown under the optimal conditions, the plants were cultivated with soil in a cell plant tray (65 × 65 × 55 mm of each cell size). For cultivation in a greenhouse at University of Tsukuba (Tsukuba, Japan), seedlings were grown with the soil and tray, and 1000-fold diluted Hyponex (HYPONeX JAPAN Co., Ltd.) was supplied after the flowering stage twice a week. For the yield tests conducted at the greenhouse from June to September in 2014, WT, homozygous *pro* and homozygous *pro-2* plants were grown in 15-cm pots and the temperature during cultivation was recorded using a data logger TR-72ui (T&D). In both the optimal and greenhouse conditions, flowers were spontaneously self-pollinated, except when otherwise stated, and the presence of seeds within fruit was examined. All selected flowers for the parthenocarpic test or for crossing with other genotypes were emasculated one day before anthesis to prevent self-pollination.

### Sequencing and genotyping of the mutation

Genomic DNA of four *pro-2* (two M_3_ and two M_4_ generation) and two WT plants was extracted from the fresh leaves using a Maxwell 16 DNA purification kit according to the manufacturer’s protocol (Promega). The extracted DNA was used as a template of PCR using specific primers (SlDELLA-orf-forward: ATGAAGAGAGATCGAGATCGAG; SlDELLA-orf-reverse: TTACAACTCGACTTCTCCGGC) and Ex Taq DNA polymerase (TaKaRa) to amplify the coding region of SlDELLA/PROCERA (Solyc11g011260). The *SlDELLA* PCR products were subjected to agarose gel electrophoresis, and then fragments were purified by the Wizard SV Gel and PCR Clean-Up system kit (Promega). The purified PCR products were used as a template for sequencing using PRISM 3130 (Applied Biosystems) coupled with BigDye Terminator v3.1 Cycle Sequencing Kit (Life technologies).

To determine the genotype of the *SlDELLA* mutation allele (c1669t) found in *pro-2*, primers for the derived cleaved amplified polymorphic sequence (dCAPS) analysis were designed (forward: GATTTGGTGATGTCGGAGGTTTATT; reverse: AGCTTCCAGGCGGAGGTAGCTTTAA) by a web-based program (dCAPS Finder 2.0; http://helix.wustl.edu/dcaps/)^47^ to yield a 284 bp PCR product. Genomic DNA was extracted as described above and used as a template of PCR using GoTaq Green Master Mix (Promega). Thermal cycling conditions were: 95 °C for 2 min followed by 35 cycles of 30 s at 95 °C, 30 s at 54 °C and 20 s at 72 °C, and finally 5 min at 72 °C. The PCR products were digested with DraI (New England Biolabs) and then electrophoresed using 2% agarose gel. DraI can digest only the product derived from the mutant allele, resulting in 261- and 23-bp fragments. The DraI-digested product of heterozygous allele plants thus resulted in three fragments, consisting of the 261-bp, the 23-bp and the 284-bp fragment derived from non-digested WT allele.

### Creation of transgenic plants

For transgenic complementation experiments of the *pro-2* mutation, the *SlDELLA* coding genomic region, which includes 2502 bp of upstream promoter sequences from the translation initiation site (without a stop codon) was synthesized and cloned into *Hin*dIII-*Sac*I sites of 35S:MIR:HSP^48^ using the In-Fusion technique (TAKARA). The destination construct (pSlDELLA::SlDELLA^WT^) was designated to express WT *SlDELLA*, C-terminally tagged with HA and MYC, under the control of its native promoter. For the creation of transgenic plants expressing the aberrant form of the *SlDELLA* found in *pro-2*, the *SlDELLA* coding genomic region between the start and stop codons was amplified by PCR with SlDELLA-orf-forward and -reverse primers using genomic DNA extracted from *pro-2* leaves. The PCR product was cloned into the entry vector pCR8/GW/TOPO (Invitrogen) by TA cloning and then sub-cloned into the pGWB15 vector^49^ using the Gateway LR Clonase enzyme (Invitrogen). The destination construct (p35S::SlDELLA^L567F^) was designed to express the *pro-2*-form mutated *Sldella*, N-terminally tagged with HA, under the control of 35S promoter.

These complete vectors were introduced into *Agrobacterium tumefaciens* strain GV2260 by electroporation. Micro-Tom *pro-2* and WT were used as the transgenic background for the introduction of pSlDELLA::SlDELLA^WT^ and p35S::SlDELLA^L567F^, respectively. Transformation of tomato plants was performed using the transformed *A. tumefaciens*^50^. Only diploid plants were selected from the regenerated plants that survived on MS plates containing the selection antibiotic, kanamycin (100 mg L^−1^). T_2_ and T_1_ generation plants harboring the pSlDELLA::SlDELLA^WT^ and p35S::SlDELLA^L567F^, respectively, were used for further analyses. qRT-PCR was conducted^51^ using the leaves of the transgenic plants introduced with p35S::SlDELLA^L567F^ and the azygous plants. Primer pairs were designed for a region spanning across *SlDELLA*^*L567F*^ and the N-terminus HA-tag or conserved region between *SlDELLA*^*L567F*^ and endogenous *SlDELLA*, to measure the expression level of exogenous *SlDELLA*^*L567F*^ or encompassing both *SlDELLA*^*L567F*^ and endogenous *SlDELLA* transcripts, respectively. The *CAC* gene was used as a reference and the expression level was normalized to the maximum expression within the test.

### Measurement of Brix value and sugar content

Soluble solids content (Brix values) in the juice of cut fruit were measured with a Brix refractometer (Atago Co., Ltd.). The contents of hexoses were measured in the soluble fraction of an ethanol extract based on methods described previously^52–54^. Briefly, aliquots of about 20 mg fresh weight of fruit pericarp were fractionated in 96-well microplates using a Star pipetting robot (Hamilton). Soluble sugars were then measured in the supernatant by measuring the absorbance at 340 nm in an MP96 microplate reader (SAFAS), in the presence of glucose-6-phosphate dehydrogenase coupled to hexokinase, phosphoglucose isomerase and invertase, which were sequentially added to determine glucose, fructose, and sucrose contents, respectively.

### Measurement of carotenoid content

The β-carotene and lycopene contents of each fruit were measured based on the method described by Nagata and Yamashita^55^. Briefly, the frozen fruit pericarp was ground into a fine powder in liquid nitrogen. Carotenoids were then extracted with acetone–hexane (4:6, v/v), and the resulting clear supernatant was used for measurement. The absorbance values at 663 nm (A663), 645 nm (A645), 505 nm (A505) and 453 nm (A453) were measured using a spectrophotometer (ND-2000C, Thermo Fisher Scientific), and the contents of β-carotene (*C*_CAR_) and lycopene (*C*_LYC_) were calculated from the following equation:

$${C}_{{\rm{CAR}}}=0.216{{\rm{A}}}_{663}-1.22\,{{\rm{A}}}_{645}-0.304\,{{\rm{A}}}_{505}+0.452\,{{\rm{A}}}_{453}$$

$${C}_{{\rm{LYC}}}=\mbox{--}0.0458\,{{\rm{A}}}_{663}+0.204\,{{\rm{A}}}_{645}+0.372\,{{\rm{A}}}_{505}-0.0806\,{{\rm{A}}}_{453}$$

### Paclobutrazol treatment

Seeds were sterilized with 10% commercial bleach including a detergent (Kitchen Haiter, Kao) for 20 min and then rinsed with sterile water three times for 5 min each. The seeds were imbibed in sterile water with gentle rotation for 3 d. The germinated seeds with emerged root of 4–6 mm were grown in 1/2 MS medium^56^ containing 0, 1 or 10 μM paclobutrazol (PAC) (Sigma-Aldrich). The lengths of root and shoot of the grown seedlings were measured at 12 days after planting.

### Yeast two hybrid assay

Two Gateway-compatible vectors, pDEST32-HA (bait) with HA-tag and pDEST22-FLAG (prey) with 3xFLAG-tag in the upstream region of the Gateway cassette were used for the yeast two hybrid (Y2H) assay^57^. Full-length open reading frames of WT SlDELLA proteins (SlDELLA^WT^), two mutated-type (SlDELLA^L567F^, SlDELLA^V302E^) and three GID1-like proteins (SlGID1b-1, SlGID1b-2 and SlGID1ac) were cloned into the entry vector pCR8/GW/TOPO using the In-Fusion system (TaKaRa). The fragments of SlDELLA proteins were then sub-cloned into the pDEST22-FLAG vector that harbors an activation domain (AD) to generate prey vectors via the LR reaction according to the manufacturer’s instructions (Thermo Fisher Scientific). The fragments of three GID1-like proteins were sub-cloned into the pDEST32-HA plasmid that harbors the DNA-binding (DB) domain to generate bait vectors via the LR reaction. Yeast strain PJ69-4A was transformed with a total of 16 possible combinations of DB and AD destination vectors to assess GAL4-based protein-protein interactions (PPIs)^58^. Cells were grown on SD-Trp-Leu (SD-WL) plates, which lack Trp and Leu, at 30 °C for 4 d. Individual colonies were picked from the plates, streaked to another SD-WL plate and grown at 30 °C for 3 d. Grown cells were suspended in a 0.9% NaCl solution and diluted (optical density at 600 nm = 0.05). Diluted cultured cells were spotted on following plates: (1) SD-WL plates, (2) SD-WLHA plates that additionally lack His and Ade, (3) SD-WLH plates that contain Ade but lack His with 0, 20 or 50 mM 3-amino-1,2,4-triazole (3-AT) and (4) SD-WLH + GA that additionally contains 100 μM GA_3_. Spotted yeast cells were grown at 30 °C for 3–5 d to evaluate PPIs. The 3-AT was used as a competitive inhibitor of reporter HIS3, an enzyme required for His biosynthesis and the growth of yeast cells on the selection medium in this system.

## Electronic supplementary material

Supplementary information
