# Supplementary material for: Identification and functional study of a mild allele of SlDELLA gene conferring the potential for improved yield in tomato
Source: Sci Rep. 2018 Aug 13;8:12043. doi: 10.1038/s41598-018-30502-w (PMC6089951; doi:10.1038/s41598-018-30502-w)
Supplement: Supplementary file 1 — Supplementary information [file 41598_2018_30502_MOESM1_ESM.pdf]

# Supplementary information

## Identification and functional study of a mild allele of *SlDELLA* gene conferring the potential for improved yield in tomato

Yoshihito Shinozaki[1,2,5], Kentaro Ezura[1,2,5], Jianhong Hu[3], Yoshihiro Okabe[1], Camille Bénard[4], Duyen Prodhomme[4], Yves Gibon[4], Tai-ping Sun[3], Hiroshi Ezura[1] & Tohru Ariizumi[1*]

[1]Faculty of Life and Environmental Sciences, University of Tsukuba, Tsukuba, Ibaraki 305-8572, Japan
[2]Research Fellow of Japan Society for Promotion of Science (JSPS), Kojimachi, Tokyo 102-0083, Japan
[3]Department of Biology, Duke University, Durham, North Carolina 27708, USA
[4]UMR 1332 Biologie du Fruit et Pathologie, INRA, Univ. Bordeaux, Villenave d'Ornon, F-33883, France

[5]These authors contributed equally to this work.
[*]Correspondence should be addressed to T.A.
(ariizumi.toru.ge@u.tsukuba.ac.jp)

**Table S1** Comparison of plant height between different *SlDELLA* alleles.

| Population | Seven-week-old plant height (cm) of each genotype[z] | | |
| --- | --- | --- | --- |
| | WT/HO | HE | Mut/HO |
| WT[y] | 8.4 ± 1.3a | – | – |
| TOMJPE2753[y] | – | – | 13.2 ± 2.3b |
| $F_2$ (TOMJPE2753 × WT)[x] | 8.0 ± 0.4a | 8.8 ± 0.5a | 11.8 ± 0.4b |

[z]Averaged height of plants with WT homozygous (WT/HO), heterozygous (HE) and mutant homozygous (Mut/HO) allele for the *SlDELLA*. [y]Obtained from five plants. [x]Obtained from13, 18 and 13 plants for WT/HO, HE and Mut/HO, respectively. Different letters indicate significant differences ($P < 0.05$; Tukey–Kramer test).

**Table S2** Comparison of parthenocarpic rate between different *SlDELLA* alleles.

| Population | Parthenocarpic rate (%) of each genotype$^{z}$ | | |
|---|---|---|---|
| | WT/HO | HE | Mut/HO |
| WT$^{y}$ | 14.9± 6.1a | – | – |
| TOMJPE2753$^{y}$ | – | – | 75.3± 3.2c |
| $F_2$ (TOMJPE2753 × WT)$^{x}$ | 18.4± 6.1ab | 31.4± 6.1b | 75.7± 5.1c |

$^{z}$Averaged parthenocarpic rate of plants with WT homozygous (WT/HO), heterozygous (HE) and mutant homozygous (Mut/HO) allele for the *SlDELLA*. $^{y}$Obtained from five plants. $^{x}$Obtained from13, 18 and 13 plants for WT/HO, HE and Mut/HO, respectively. Different letters indicate significant differences ($P < 0.05$; Tukey–Kramer test).

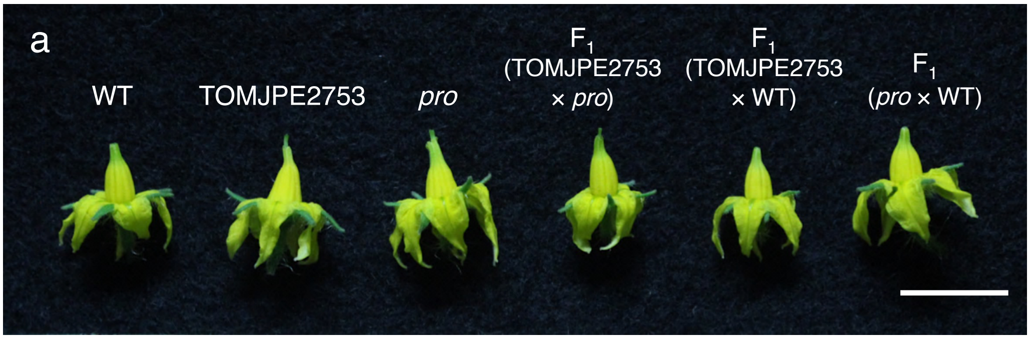


b

| Genotypes | Parthenocarpic fruit set efficiency<br>% (developed fruit/emasculated flowers) |
|---|---|
| TOMJPE2753 | 75% (15/20) |
| *pro* | 81% (25/31) |
| $F_1$ (TOMJPE2753 × *pro*) | 90% (9/10) |
| WT | 5% (1/22) |
| $F_1$ (TOMJPE2753 × WT) | 8% (1/13) |
| $F_1$ (*pro* × WT) | 20% (5/20) |

The numbers of developed mature fruit out of emasculated flowers are shown.

**Fig. S1** Tests of allelism between TOMJPE2753 and *pro*. (a) Allelism assessed by stylar elongation. Bar = 1 cm. (b) Allelism assessed by parthenocarpic rate.

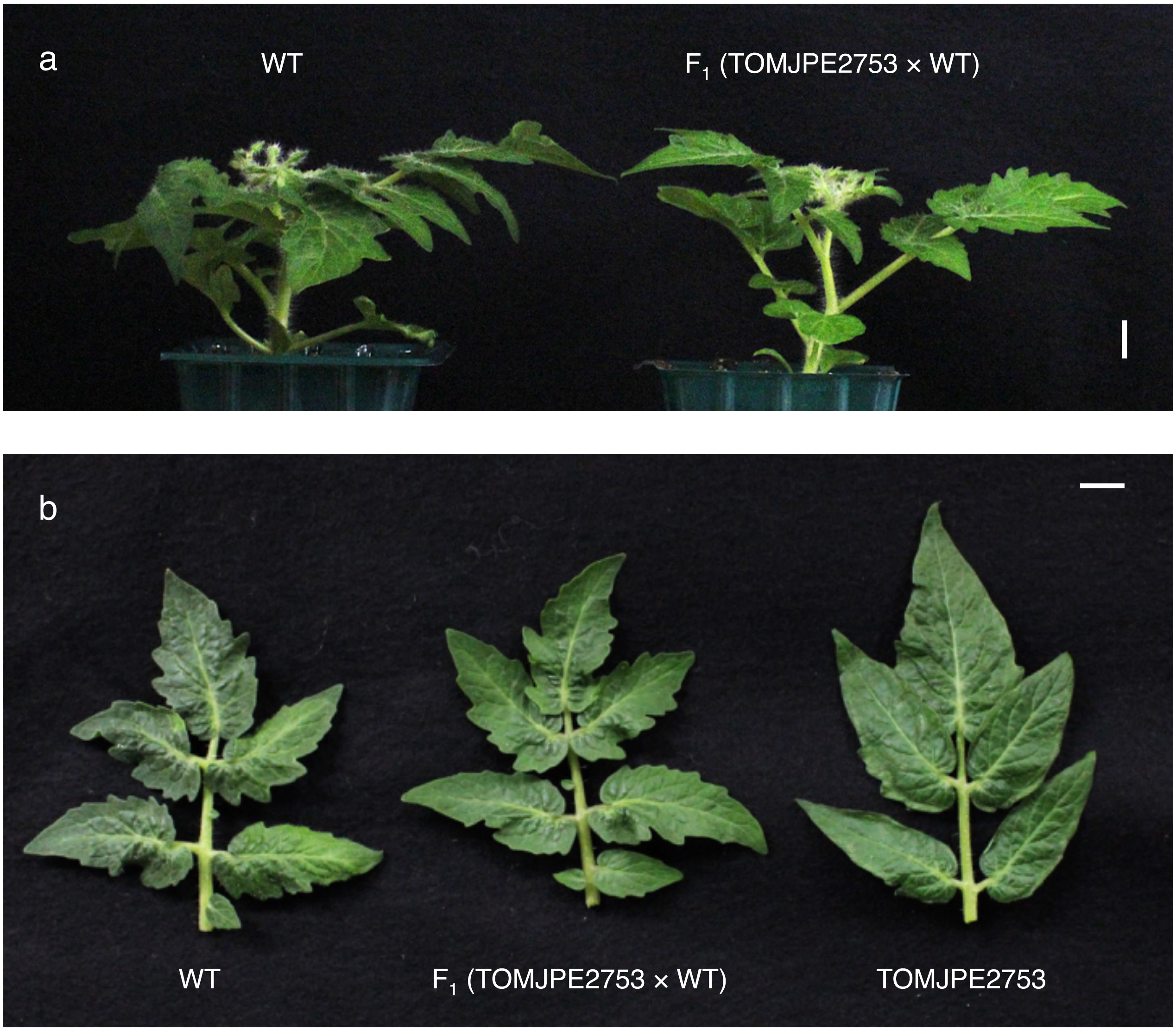


**Fig. S2** Representative $F_1$ progeny of a cross between TOMJPE2753 and the WT. (a) Six-week-old plants. (b) Leaflets of the 6th node. Bars = 1 cm.

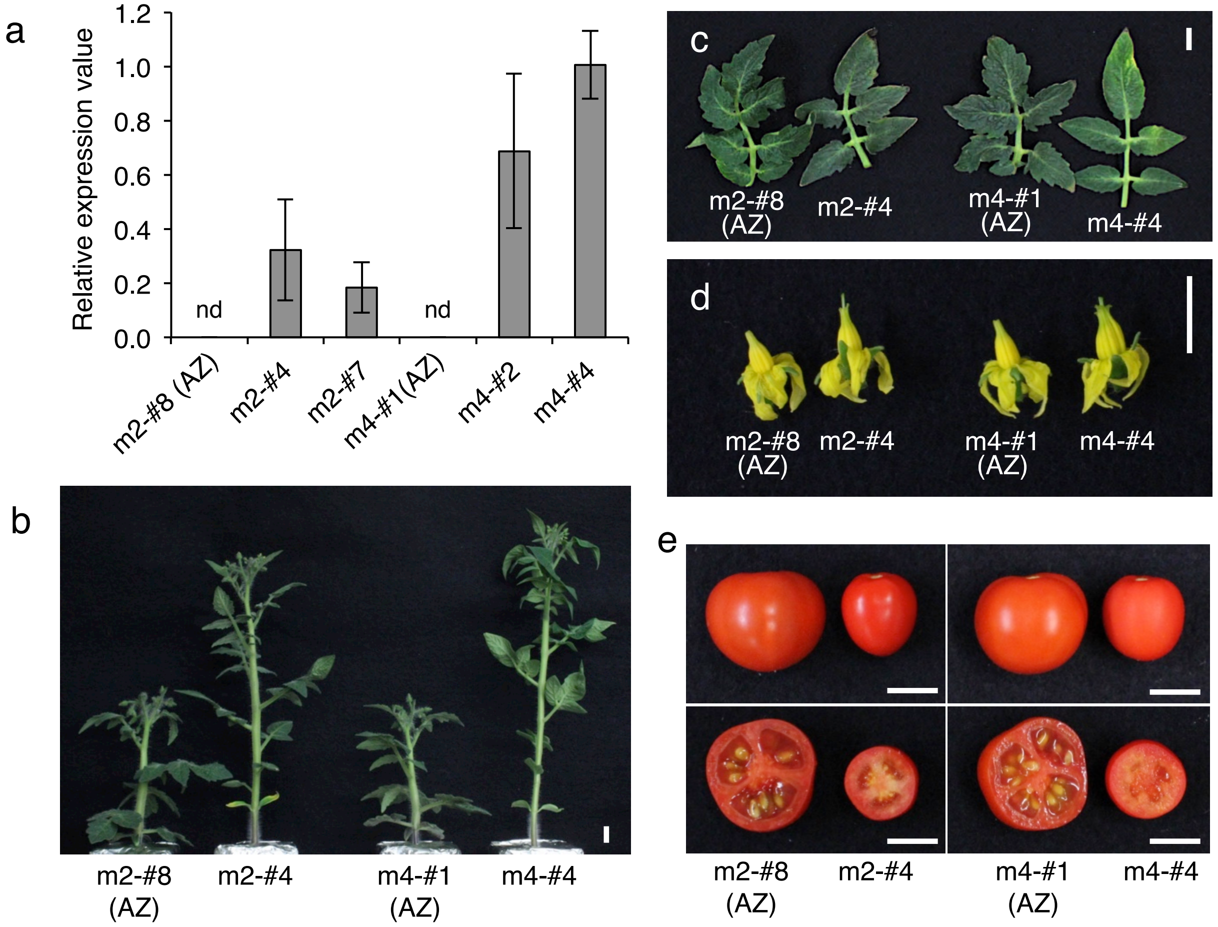


f

| Plants | Parthenocarpic fruit set efficiency<br>% (developed fruit/emasculated flowers) |
|---|---|
| p35S::SlDELLA$^{L567F}$-m2-#8 (AZ) | 0% (0/10) |
| p35S::SlDELLA$^{L567F}$-m2-#4 | 100% (10/10) |
| p35S::SlDELLA$^{L567F}$-m2-#7 | 100% (10/10) |
| p35S::SlDELLA$^{L567F}$-m4-#1 (AZ) | 0% (0/10) |
| p35S::SlDELLA$^{L567F}$-m4-#2 | 100% (10/10) |
| p35S::SlDELLA$^{L567F}$-m4-#4 | 100% (10/10) |

**Fig. S3** Effect of *SlDELLA$^{L567F}$* expression on vegetative and reproductive development in a WT background. (a) Relative expression levels of *SlDELLA* derived from exogenous *SlDELLA$^{L567F}$* transcripts in the leaf of transformed plants were analyzed by qRT-PCR. *CAC* gene was used as a reference and the expression level was normalized to the maximum expression within the test. Values are mean ± SE. nd, not detected. Representative pictures of (b) six-week-old plants, (c) leaflets of the 6th node, (d) flowers and (e) fruit of transgenic and nontransgenic azygous plants. In panel e, pollinated azygous fruit and parthenocarpic fruit in transgenic lines are shown. (f) Parthenocarpic rate in transgenic lines. Two transgenic plants (m2-#4 and m2-#7) from line m2 and two transgenic plants (m4-#2 and m4-#4) from line m4 were used along with their corresponding azygous siblings m2-#8 and m4-#1, respectively. AZ, azygous. Bars in b-e = 1 cm.

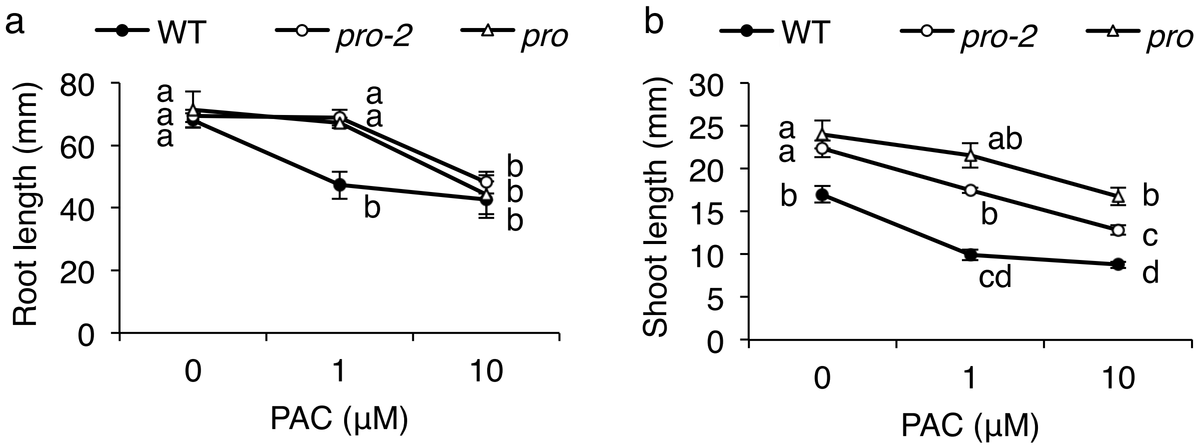


**Fig. S4** Comparison of PAC sensitivity among WT, *pro*-2 and *pro* seedlings. (a) Root and (b) shoot length at 12 days after starting PAC treatment. Values are mean ± SE (n = 7).

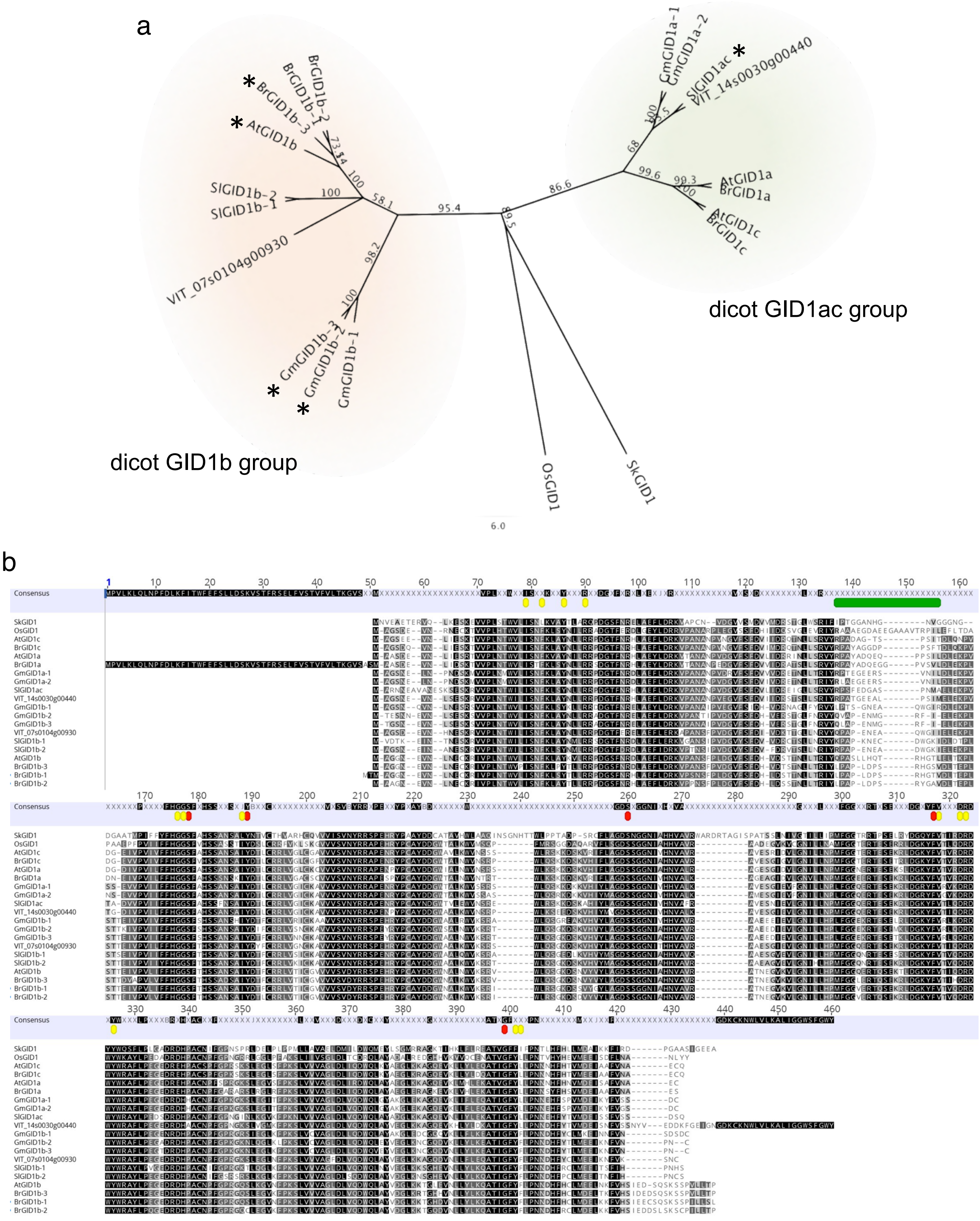


**Figure S5** GA receptor GIBBERELIN INSENSITIVE DWARF (GID1) proteins in tomato. (a) Phylogenetic tree of full-length of GID1 proteins of tomato and other species. Asterisks represent members showed GA-independent interaction with DELLA proteins in this study or previous studies. (b) The alignment of full-length of GID1 proteins of tomato, Arabidopsis, rice. closed circle represents residues to bind GA; hydrogen bonds (Red), other bonds (Yellow). Green box represents the loop region related to GA-independent GID1-DELLA interaction. At, Arabidopsis; Os, rice; Gm, Soybean; Br, Brassica rapa; VIT, Grape; Sk, *Selaginella kraussiana*.

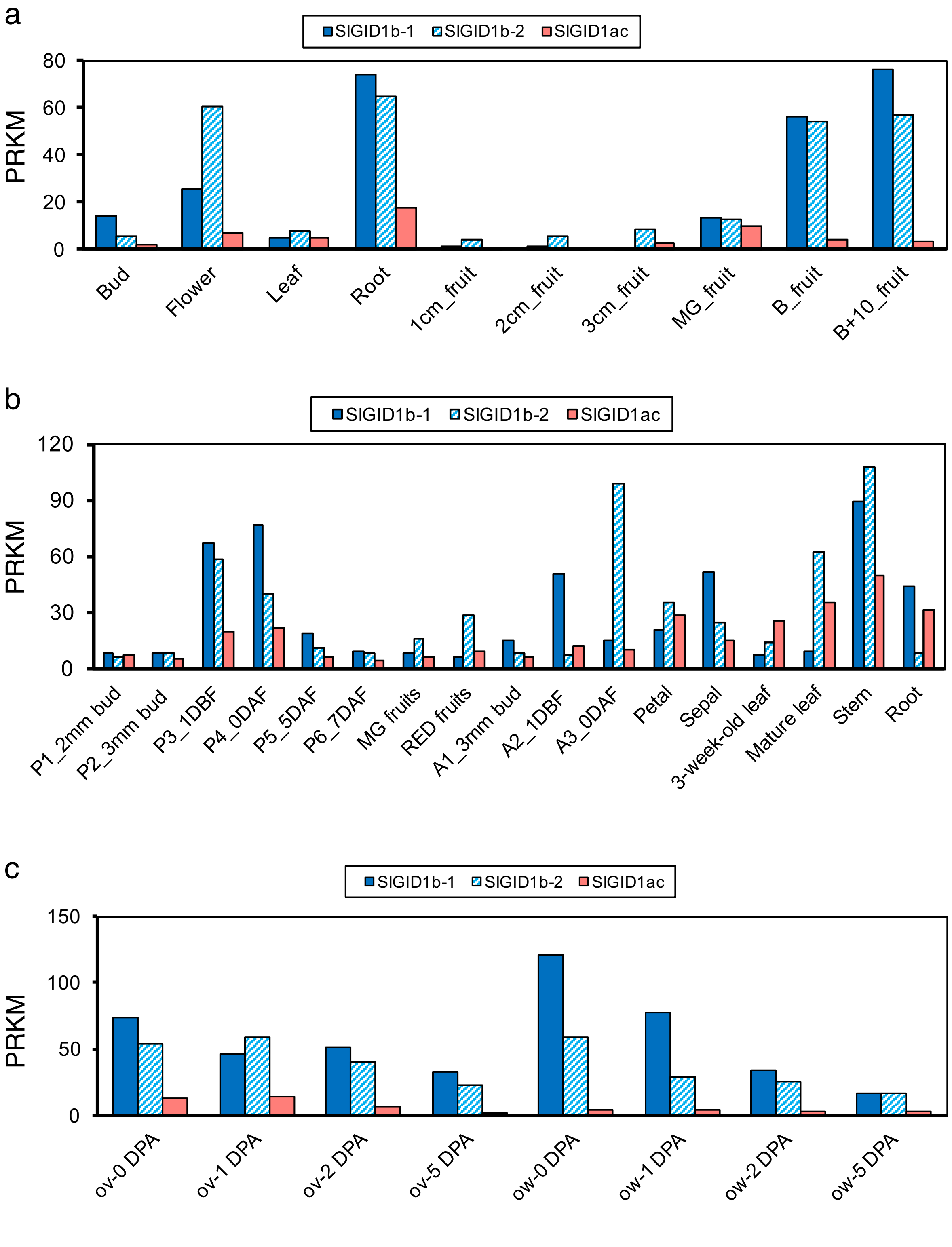


**Figure S6** Expression of *SlGID1* family genes in various tomato tissues. (a) The gene expression of *SlGID1s* in various tissue of tomato cultivar 'Heinz 1706' from Tomato genome consortium (2012). (b) The gene expression of *SlGID1s* in various tissue of tomato cultivar 'Micro-Tom' from Ezura *et al*. (2017). (c) The gene expression of *SlGID1s* in the ovary tissues of tomato cultivar 'Moneymaker' from Zhang *et al*. (2016). RPKM, reads per kilobase of transcript per million of sequenced reads; MG, mature green; B, Breaker; P1-6, pistil samples; A1-3, anther samples; ov, ovule; ow, ovary wall/pericarp; DBF, day before flowering; DAF, days after flowering; DPA, days post anthesis.

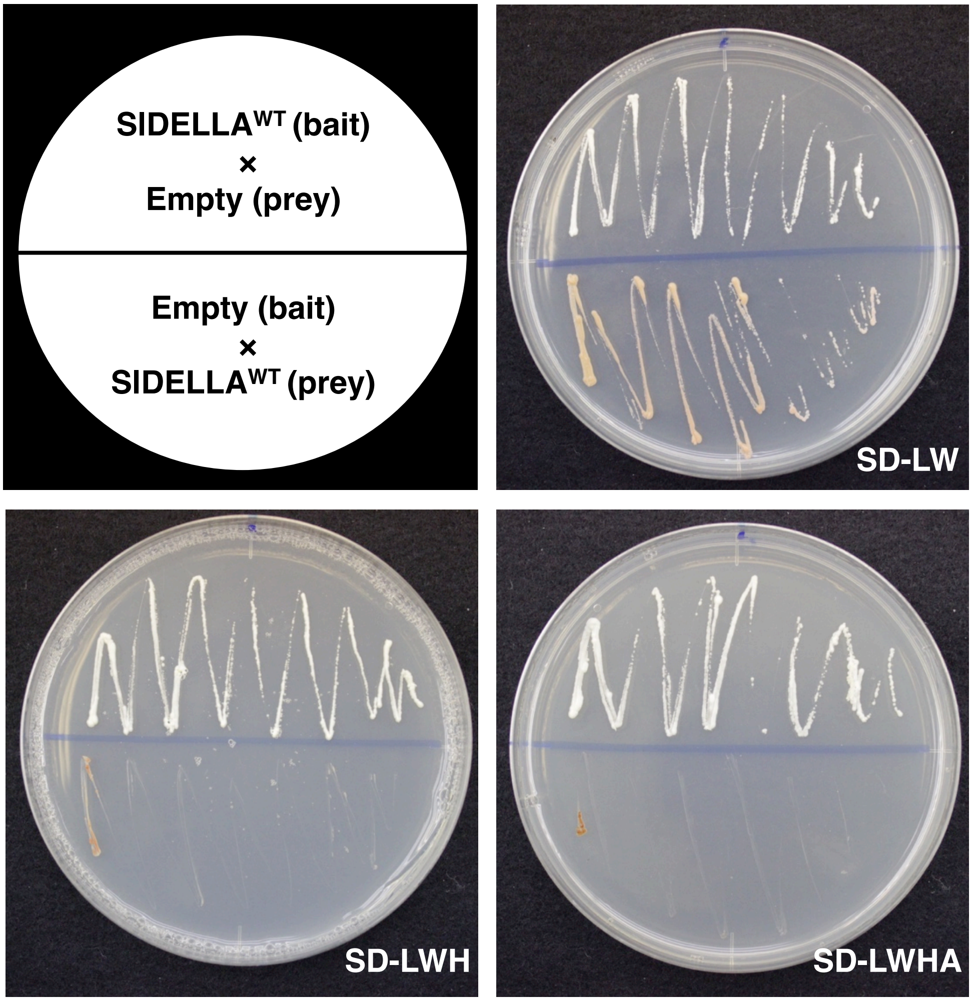


**Fig. S7** SlDELLA showed autoactivation in yeast two hybrid assay when it fused with DNA binding domain as a bait. The yeast cells transformed with the full-length SlDELLA as a bait construct grow on the selective plates (SD-LWH and SD-LWHA) without an interacting partner in prey construct.
